# Costunolide-Induced Apoptosis via Promoting the Reactive Oxygen Species and Inhibiting AKT/GSK3β Pathway and Activating Autophagy in Gastric Cancer

**DOI:** 10.3389/fcell.2021.722734

**Published:** 2021-11-12

**Authors:** Cuixiang Xu, Xiaoyan Huang, Xiaohua Lei, Zhankui Jin, Min Wu, Xiao Liu, Yubin Huang, Xiangrong Zhao, Yue Xiong, Jingying Sun, Xianglong Duan, Jianhua Wang

**Affiliations:** ^1^Shaanxi Provincial Key Laboratory of Infection and Immune Diseases, Shaanxi Provincial People’s Hospital, Xi’an, China; ^2^Center for Energy Metabolism and Reproduction, Shenzhen Institutes of Advanced Technology, Chinese Academy of Sciences, Shenzhen, China; ^3^Department of Orthopedics, Shaanxi Provincial People’s Hospital, Xi’an, China; ^4^Department of Research, Shaanxi Provincial People’s Hospital, Xi’an, China; ^5^Department of Graduate School, Xi’an Medical University, Xi’an, China; ^6^Second Department of General Surgery, Shaanxi Provincial People’s Hospital, Xi’an, China

**Keywords:** costunolide, apoptosis, autophagy, ROS, Akt/GSK3β, gastric cancer

## Abstract

**Objective:** Costunolide (Cos) is a sesquiterpene lactone extracted from chicory. Although it possesses anti-tumor effects, the underlying molecular mechanism against gastric cancer cells remains unclear. This study aimed to explore the effect and potential mechanism of Cos on gastric cancer.

**Methods:** The effect of Cos on HGC-27 and SNU-1 proliferation was detected by CCK-8 and clone formation assay. The changes in cell apoptosis were determined using Hoechst 33258 and tunel staining. The morphology of autophagy was analyzed by autophagosomes with the electron microscope and LC3-immunofluorescence with the confocal microscope. The related protein levels of the cell cycle, apoptosis, autophagy and AKT/GSK3β pathway were determined by Western blot. The anti-tumor activity of Cos was evaluated by subcutaneously xenotransplanting HGC-27 into Balb/c nude mice. The Ki67 and P-AKT levels were examined by immunohistochemistry.

**Results:** Cos significantly inhibited HGC-27 and SNU-1 growth and induced cell cycle arrest in the G2/M phase. Cos activated intrinsic apoptosis and autophagy through promoting cellular reactive oxygen species (ROS) levels and inhibiting the ROS-AKT/GSK3β signaling pathway. Moreover, preincubating gastric carcinoma cells with 3-methyladenine (3-MA), a cell-autophagy inhibitor, significantly alleviated the effects of Cos in inducing cell apoptosis.

**Conclusion:** Cos induced apoptosis of gastric carcinoma cells via promoting ROS and inhibiting AKT/GSK3β pathway and activating pro-death cell autophagy, which may be an effective strategy to treat gastric cancer.

## Introduction

Gastric cancer (GC), one of the most common malignancies worldwide, is the third leading cause of cancer deaths worldwide (Bray et al., 2018), with more than half of the cases occurring in East Asia especially in China, Japan, and South Korea (Rahman et al., 2014). In China, gastric cancer is among the most common malignancies, and its number of new cases accounts for 46% of the global incidence (Hamashima, 2014; Zong et al., 2016; Wang K. et al., 2020). Gastric cancer is often diagnosed late and is composed of several subtypes with distinct biological and molecular properties. Therefore, 25–50% of gastric cancer cases metastasized during disease progression (Johnston and Beckman, 2019). Currently, surgery is the preferred treatment for patients against gastric cancer, and chemotherapy remains the primary option for patients with advanced gastric cancer (Cunningham et al., 2006). However, more than half of the gastric cancer patients undergoing radical resection developed local recurrence or distant metastasis, and the prognosis is generally poor (Efferth et al., 2008). In addition, another important problem in tumor chemotherapy is the development of drug resistance and side effects (Turner et al., 2012), so that most patients with gastric cancer share a poor quality of life, with a survival time of less than 5 years in a majority of cases (Suzuki et al., 2016). Therefore, novel drugs against gastric cancer with low toxicity and high potency need to be developed urgently in the clinic.

Plants have long been regarded as a rich source of natural products with a broad range of bioactivities, and numerous studies have identified natural products with anti-cancer activities (Zhang J. Y. et al., 2016; Lin et al., 2017; Kang et al., 2019; Liu et al., 2019). Costunolide (Cos) is a natural sesquiterpene lactone extracted from various medicinal plants (Cao et al., 2016), including Saussurea, costus, and chicory (Garayev et al., 2017). Accumulating evidence has demonstrated multiple pharmacological activities of Cos, including anti-inflammatory, anti-allergic, and anti-microbial effects (Duraipandiyan et al., 2012; Park et al., 2016; Lee et al., 2018). Recent studies have found that Cos possesses anti-cancer effects against human gastric adenocarcinoma, prostate cancer, liver cancer, bladder cancer, and esophageal cancer, and promotes apoptosis of a variety of cancer cells (Rasul et al., 2013; Hua et al., 2016a; Chen et al., 2017; Mao et al., 2019; Yan et al., 2019). However, the molecular mechanism underlying the effects of Cos against gastric cancer cells has yet to be elucidated.

Programmed cell death (PCD) plays an important role in cancer pathogenesis and treatment, including apoptosis, autophagy, and programmed necrosis and other mechanisms. The form of type I PCD is called apoptosis, with characteristics of cell membrane blebbing, cell shrinkage, and chromatin condensation (Burgess, 2013), which occurs in two main classical pathways: (1) the external pathway, stimulated by the activation of the death receptor ligand system; and (2) the internal pathway, caused by the change of mitochondrial membrane permeability, the formation of the apoptosome, and the release of apoptosis-related proteins. The form of type II PCD is termed autophagy, with characteristics of autophagosomes and autophagolysosomes appearing in the cytoplasm, digested eventually and degraded by their own lysosomes, causing cell death (Al-Bari and Xu, 2020).

Reactive oxygen species (ROS) plays a vital role as a “second messenger” in the intracellular signal cascade, controlling the growth, proliferation, migration, and apoptosis or PCD of cancer cells. An excessive amount of ROS caused oxidative damage in the mitochondria of cancer cells to interfere with cell signaling pathways, such as AKT (protein kinase B, PKB)/glycogen synthase kinase-3β (GSK3β) signaling pathway. AKT phosphorylation and the regulation of downstream effector molecules GSK3α/β play a key role in regulating cell survival, growth, and metabolism (Al-Bari and Xu, 2020).

In this study, we investigated the effect of Cos on the proliferation, cell cycle, apoptosis, and autophagy of gastric cancer GC cell lines both *in vitro* and *vivo*. The results showed that Cos inhibited HGC-27 and SNU-1 cell growth and induced apoptosis and autophagy via the ROS-AKT/GSK3β pathway and induced apoptosis through activating pro-death autophagy, which provides experimental support and a theoretical basis for further research on the role of Cos in gastric cancer treatment.

## Materials and Methods

### Experimental Reagents

Gastric carcinoma cell lines (HGC-27) (cat. No. CL-0107) and (SNU-1) (cat. No. CL-0474), normal human gastric epithelial cells (GES-1) (cat. No. CL-0563), and fetal bovine serum (FBS) were purchased from Wuhan Procell Life Technology, Wuhan, China. RPMI-1640 medium was purchased from Gibco (Thermo Fisher Scientific, Carlsbad, CA, United States). The Hoechst 33258 staining solution (C1017), TUNEL Apoptosis Assay Kit (C1088), and BCA Protein Assay Kit (P0012) were procured from Beyotime Institute of Biotechnology, Shanghai, China. Rabbit anti-human Cyclin B1 (1:1,000, cat. No. 12231S), Rabbit anti-human cell division cyclin 25 homolog C (Cdc25c) (1:1,000, cat. No. 4866S), Rabbit anti-human Cdk1(1: 1,000, cat. No. 77055S), Rabbit anti-human Caspase 3 (1: 1,000, cat. No. 9662S), Rabbit anti-human Bcl-2(1:1,000, cat. No. 4223S), Rabbit anti-human Bax (1: 1,000, cat. No. 2774S), Rabbit anti-human Bak (1:1,000, cat. No. 12105S), Rabbit anti-human PARP (1:1,000, cat. No. 9532S), Rabbit anti-human Caspase 8 (1:1,000, cat. No. 4790S), Rabbit anti-human death receptor-4 (DR4) (1:1,000, cat. No. 42533S), Rabbit anti-human death receptor-5 (DR5) (1:1,000, cat. No. 69400S), Rabbit anti-human Fas ligand (FasL) (1:1,000, cat. No. 68405S), Rabbit anti-human Fas (1: 1,000, cat. No. 4233S), Rabbit anti-human microtubule-associated protein1 light chain3B (LC3B) (1:1,000, cat. No. 3868S), Rabbit anti-human IRE1α (1:1000, cat. No. 3294S), Rabbit anti-human p62 (1:1,000, cat. No. 5114S), Rabbit anti-human Beclin (1:1,000, cat. No. 3495S), Rabbit anti-human PARP (1:1,000, cat. No. 9532S) antibody, rabbit anti-human AKT (1:1,000, cat. No. 9272S) antibody, rabbit anti-human phosphor-Akt (1:2,000, cat. No. 4060S) antibody, rabbit anti-human GSK3β (1:1,000, cat. No. 9315S) antibody, rabbit anti-human phosphor-GSK3β (Ser 9) (1:1,000, cat. No. 9322S) antibody, and rabbit anti-human glyceraldehyde-3-phosphate dehydrogenase (GAPDH) (1:1,000, cat. No.5174S) antibody were obtained from Cell Signaling Technology, Cambridge, MA, United States. Rabbit anti-mouse Ki-67 (1:1,000, cat. No. ab16667) was obtained from Abcam, Cambridge, United Kingdom. A horseradish peroxidase (HRP)-labeled goat anti-rabbit Immunoglobulin G (IgG) antibody (1:2,000, cat. no. CW0103) was purchased from CWbio, Beijing, China. Cell Counting Kit-8 kits (CCK-8), ROS detection kits (S0033S), Annexin V-fluorescein isothiocyanate (FITC) apoptosis detection kits (C1062M), and cell cycle detection kits (C1052) were purchased from Beyotime. D-luciferin (122799) was from Perkin Elmer, Waltham, MA, United States. NAC (HY-B0215), SC79 (HY18749), 3-Methyladenine (3-MA) (HY-19312) were from MedChemExpress, Monmouth Junction, NJ, United States. 4% polyformaldehyde was from Solarbio, Beijing, China.

### Cell Culture

HGC-27 and SNU-1 were cultured in Roswell Park Memorial Institute 1640 (RPMI 1640) (containing 100 μg/ml streptomycin and 100 IU/ml penicillin) supplemented with 100 ml/L FBS and kept at 37°C with 5% CO_2_ atmosphere.

### Cell Proliferation Assay and Observation of Cell Morphology

HGC-27 and SNU-1 that are in the logarithmic growth phase were collected and inoculated into a 96-well plate at 5 × 10^3^ cells/well, cultured overnight at 37°C; then HGC-27 and SNU-1 were treated with Cos at different concentrations (0, 2.5, 5, 10, 20, 40, 80, and 160 μmol/L) in FBS-free RPMI 1640 for 24 and 48 h, and cell proliferation was detected by the Cell Counting Kit-8 assay. We added 10 μl of CCK-8 reagent to the cells in each well and incubated them at 37°C for 4 h; optical density values were measured with a microplate reader at 450 nm. After the half-maximum inhibitory concentration (IC50) was determined, the cells in four different Cos concentrations were selected according to the IC50, observed, and photographed under inverted light microscopy (Leica, DMIL, Germany × 200). The cells in five microscope fields of view were randomly selected for counting to evaluate the cell viability in each group.

### Colony Formation Assay

HGC-27 and SNU-1 were seeded into the 60 mm dish at a density of 500 cells/well and cultured into RPMI 1640 containing 10% FBS for 24 h, then treated with various concentrations of Cos (0, 10, 20, and 40 μM). The treated cells were resuspended in RPMI 1640 containing 10% FBS and cultured in 5% CO_2_ at 37°C for 15 days to form colonies. After the dish was washed with PBS, the colonies were fixed with 4% polyformaldehyde at room temperature then dyed with 1% crystal violet for 30 min at room temperature. Colonies comprising 50 cells or more were counted by microscope (Leica Microsystems, Wetzlar, Germany) as previously described (Chen et al., 2016). Each experiment was done thrice in this study. Colony formation rate = the number of each treatment/the number of control × 100%.

### Hoechst 33258 Staining

HGC-27 and SNU-1 were seeded into 12-well plates, cultured for 24 h, then treated with 0, 10, 20, and 40 μM Cos for 24 h. The adherent cells were washed twice with PBS, then stained with Hoechst 33258 (Beyotime) for 5 min at room temperature in the dark. After being washed twice, the blue-stained nucleus was observed under the BX41 fluorescence microscope (Olympus, Tokyo Japan; amplification: × 400). The nucleus of living cells presents diffuse and uniform fluorescence, and the characteristic of apoptotic cells was that the nucleus or cytoplasm presents dense granular and clumpy fluorescence. Images were captured to quantitatively analyze via Image Pro Plus analysis software 6.0 (Media Cybernetics Inc., Rockville, MD, United States).

### Tunel Staining

The apoptosis of GC cells and animal tumors were evaluated via the Tunel Apoptosis Assay Kit (Beyotime). Firstly, the cell samples and paraffin-embedded tissue sections (4 μm thick) were treated by protein kinase K and 3% H_2_O_2_, respectively, and incubated with Tunel detection solution (the component of Tunel staining kit) for 1 h at 37°C, then incubated with Streptavidin-HRP working solution. At last, the DAB solution was added and the samples were observed and photographed under the BX41 fluorescence microscope (Olympus Corporation; amplification: × 400). Images were captured to quantitatively analyze the apoptosis of cells via Image-Pro Plus analysis software 6.0 (Media Cybernetics). The number of apoptotic cells and the total number of cells were counted, and the proportion of apoptosis was calculated. Apoptosis cell proportion = number of positive cells/total number of cells × 100%.

### Flow Cytometry Assay

Cell cycle, apoptosis, and ROS level were measured by flow cytometry analysis. HGC-27 and SNU-1 (2.0 ml/well, 3 × 10^5^ cells/mL) were seeded and cultured into the six-well plate for 24 h. After aspiration, the cells were incubated with 2.0 ml of Cos at different concentrations (0, 10, 20, and 40 μmol/L) or treated with Cos before pretreating with NAC in FBS-free high-glucose DMEM for 24 h. The cell cycle detection kit, Annexin V-FITC apoptosis detection kit, and ROS detection kit were used for analysis according to the manufacturer’s instructions, respectively. Briefly, the collected cells were stained with 75% ethanol at 4°C overnight, propidium iodide (PI) for cell cycle analysis, and Annexin V-FITC and PI for 15 min at 37°C in a darkroom for apoptosis analysis, respectively. Then they were incubated with 2′,7′-dichlorodihydrofluorescein diacetate (DCFH-DA) for 15 min at 37°C in a darkroom for ROS level analysis. The cells were analyzed via flow cytometry (BD FACSCalibur; Becton Dickinson, San Jose, CA, United States).

### Western Blot Analysis

The levels of cell cycle-related protein (Cyclin B1, Cdc25c, Cdk1), intrinsic apoptosis-related proteins (Caspase 3, Bak, Bax, Bcl2, PARP), extrinsic apoptosis-related proteins (caspase 8, DR4, Fas, FasL), autophagy-related proteins (LC3B, beclin-1, p62), and signaling pathway-related proteins (AKT, P-AKT, GSK3β, and P-GSK3β) in HGC-27, SNU-1 were analyzed by Western blot analysis. Briefly, the protein of GC cell lines HGC-27 and SNU-1 was extracted with radioimmunoprecipitation assay (RIPA) lysis buffer containing protease inhibitors on ice, and quantified using the BCA Protein Assay Kit. The protein bands were separated by sodium dodecyl sulfate-polyacrylamide gel electrophoresis (SDS-PAGE) and transferred to nitrocellulose membranes. After being blocked with 5% bovine serum albumin (BSA) in phosphate-buffered saline with Tween (PBST) for 1 h, the membranes were incubated with primary antibodies at 4°C overnight, then incubated with HRP-conjugated secondary antibodies for 1 h at room temperature. The SuperSignal ELISA Femto Substrate was added onto the membranes in a darkroom and was subsequently exposed to x-ray films. The intensity of the Western bands was determined by Image J software version 1.46 [National Institutes of Health (NIH), Bethesda, MD, United States].

### Immunofluorescence

The slides with the climbed cells in the culture plate were fixed with 4% paraformaldehyde and permeabilized with 0.5% Triton X-100 for 20 min. After being blocked with BSA, the cells were incubated with the LC3B primary antibody overnight at 4°C. At last, they were incubated with Alexa-Fluor 488-conjugated secondary antibodies in 1% bovine serum at 37°C for 1 h in the dark. Nuclei were counterstained with DAPI for 15 min in the dark. Images were photographed via a confocal laser scanning microscope (OLYMPUS FV3000; Olympus Corporation, Center Valley, PA, United States; amplification: × 1000), and endogenous LC3 puncta formation were analyzed using the FV10-ASW viewer software ver. 4.2b (Olympus).

### Transmission Electron Microscopy

We harvested the cells by centrifuging at 3000 r/min for 10 min, washing twice with cold PBS, aspirating the supernatant, and fixing with 2.5% glutaraldehyde along the tube wall. Then electron microscope slices were prepared according to conventional procedures. At last, cell ultrastructure in every group was observed under the electron microscope (HITACHI, HT7700-SS, Tokyo, Japan).

### Tumor Model *in vivo*

The procedures and ethics of animal use have been reviewed and approved by the Biomedical Ethics Committee of Shaanxi Provincial People’s Hospital (The Third Affiliated Hospital of Xi’an Jiaotong University) (approval no. 2021-155). The 16 female Balb/c nude mice (5–6 weeks old, 19.5 ± 2.6 g) were from the Animal Center of Shanghai Institute of Family Planning Science (Shanghai, China) [SCXK (Hu) 2018-0006].

Firstly, the HGC-27 cells with stable expression of luciferase were constructed by lentivirus. Secondly, luciferase-positive HGC-27 cells (5 × 10^6^ cells per mouse) were injected subcutaneously into the right flank of Balb/c nude mice, when the tumor volume reached 100 mm^3^ (Festing and Altman, 2002). The mice were randomly divided into four groups (*n* = 4/group), the negative control, the positive control, and the experimental group. In the experimental group, the mice were administered intraperitoneally with 30 mg/kg and 50 mg/kg Cos, respectively, and with the same volume of dimethyl sulfoxide (DMSO) in the negative control group, with cisplatin (2 mg/kg) in the positive control group. It was injected every 3 days. The weight of the animal was analyzed every 3 days, and the length (L) and width (W) of the tumor were measured with a caliper. The volume calculation formula is:

L × (W)^2^/2 (Du et al., 2012). Thirty days later, animals were sacrificed and the dissecting tumors, heart, liver, spleen, lungs, and kidneys were for corresponding analysis.

### Hematoxylin-Eosin and Tunel Staining

The tissues (containing tumors, hearts, livers, spleens, lungs, kidneys) were fixed in 4% paraformaldehyde for 24 h, dehydrated, and embedded in paraffin. Sections 4 μm thick were stained with hematoxylin and eosin (H&E) and Tunel for morphological observation, respectively. Images were observed and photographed under the BX41 fluorescence microscope (Olympus Corporation; amplification: ×200) and quantitatively analyzed via Image-Pro Plus analysis software 6.0 (Media Cybernetics).

### Immunohistochemistry

The paraffin-embedded tissue sections (4 μm thick) were deparaffinized and rehydrated, incubated with rabbit polyclonal antibodies specific to Ki-67 and P-AKT at 4°C overnight, incubated with HRP-conjugated secondary antibody at room temperature for 2 h, and stained in hematoxylin for 3 min and observed under the BX41 fluorescence microscope (Olympus Corporation; amplification: ×200).

### *In vivo* Imaging of Balb/c Nude Mouse Tumor Model

Bioluminescence imaging (BLI) was performed using an IVIS imaging system (Perkin Elmer, Waltham, MA, United States) after 15 and 24 days after drug intervention; 100 μl PBS containing 25 mM D-luciferin (Caliper Life Sciences, Hopkinton, MA, United States) was injected intraperitoneally 10 min before luciferase detection.

### Statistical Analysis

All data were represented as mean ± SEM. The biotechnology was repeated at least three times *in vitro*. The intergroup deviations were evaluated with a one-way analysis of variance (ANOVA) implemented in the GraphPad Prism 6.0 software, with *P* < 0.05 indicating a statistically significant difference.

## Results

### Costunolide Inhibited the Proliferation and Colony Formation in GC Cells

**FIGURE 1:**
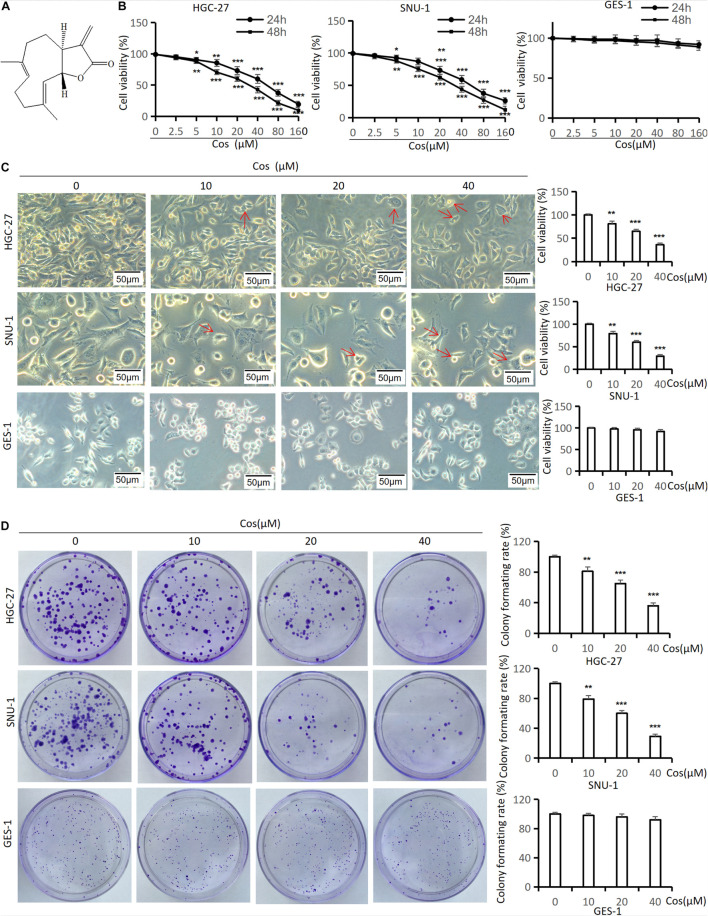
Cos inhibited GC viability and colony formation but had no effect on GES-1 cells. **(A)** Chemical structure of costunolide; molecular formula: C_15_H_20_O_2_. **(B)** The viability of HGC-27, SNU-1, and GES-1 cells treated by costunolide with different concentrations (0, 2.5, 5, 10, 20, 40, 80, 160 μmol/L) for 24 and 48 h was detected by CCK-8 assay. **(C)** HGC-27, SNU-1, and GES-1 cells were treated with different concentrations (0, 10, 20, 40 μmol/L) for 24 h; the morphology of cells was observed by inverted phase-contrast microscope (magnification: 200×) (red arrow = cell deformation) and cell viability was determined. **(D)** Cos inhibited the colony formation of GC. HGC-27, SNU-1, and GES-1 were treated with different concentrations for 15 days, stained and counted. *p < 0.05, **p < 0.01, ***p < 0.001.

CCK-8 and colony formation assay were used to analyze the effect of Cos (Figure 1A) on GC cell proliferation. As shown in Figure 1B, Cos could significantly inhibit the proliferation of HGC-27 and SNU-1 cells in a dose-dependent manner compared with that in the control group (*p* < 0.001), but the effect of Cos on normal gastric cells (GES-1) was not as sensitive as GC cells (*p* > 0.05) (Figure 1B). As shown in Figure 1B, the half-maximum inhibitory concentration (IC 50) for the two cells at 24 or 48 h is about 40 μM. Therefore, Cos concentrations of 0, 10, 20, and 40 μM were chosen in these assays. Phase-contrast microscope results showed that Cos induced shrinkage, deformation, and rupture and inhibited the proliferation in GC cell lines HGC-27 and SNU-1, but it had no effect on GES-1 (Figure 1C). In addition, colony formation assay further revealed that Cos obviously inhibited proliferation in a dose-dependent manner (*p* < 0.001) but, on GES-1, was not as sensitive as GC cells (Figure 1D).

### Costunolide Induced Cell Cycle Arrest in GC Cells

**FIGURE 2:**
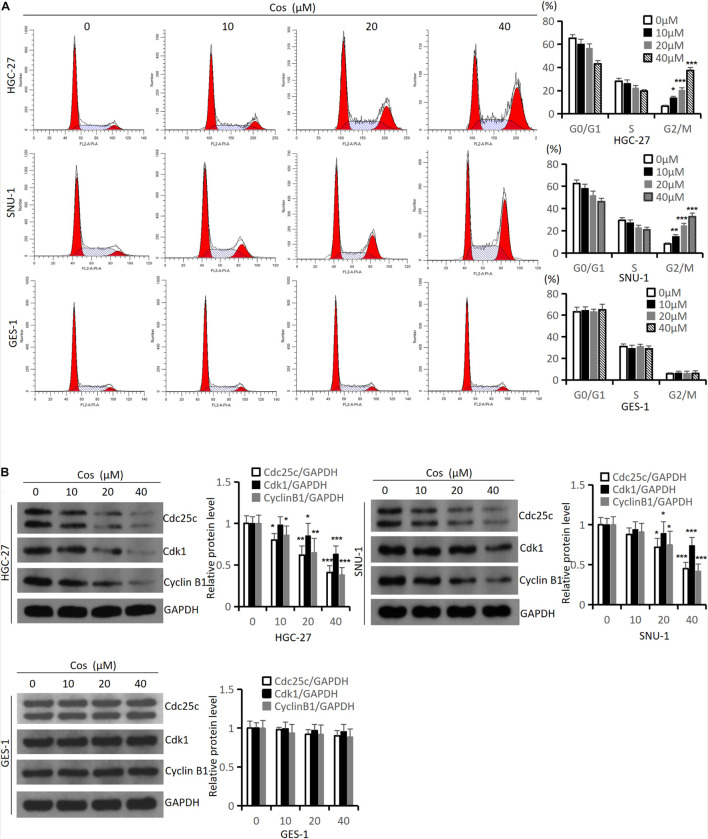
Cos induced cell cycle arrest in GC cells but had no effect on GES-1 cells. **(A)** The effect of Cos on cell cycle of HGC-27, SNU-1, and GES-1 cells was treated with different concentrations for 24 h, and determined by flow cytometry analysis. **(B)** The levels of cell cycle-associated proteins were determined by Western blot. HGC-27, SNU-1, and GES-1 were treated with indicated concentration for 24 h, then Western blot analysis was performed. Compared with control group, *p < 0.05, **p < 0.01, ***p < 0.001.

To estimate the effect of Cos on the cell cycle, we performed flow cytometry and western blot analysis in HGC-27 and SNU-1 cells. The flow cytometry results suggested that Cos significantly induced cell cycle arrest in the G2/M phase in HGC-27, SNU-1 cells with obvious dose-dependency (*p* < 0.001), but the effect of Cos on GES-1 was not as sensitive as GC cells (*p* > 0.05) (Figure 2A), and Western blot showed that the expression levels of cell cycle-related proteins (Cdc25c, Cdk1, Cyclin B1) in GC cells were significantly downregulated by Cos, especially in the 40 μM Cos group (*p* < 0.001), but the effect of Cos on GES-1 cells was not significant (*p* > 0.05) (Figure 2B).

### Costunolide Induced Apoptosis in GC Cells

**FIGURE 3:**
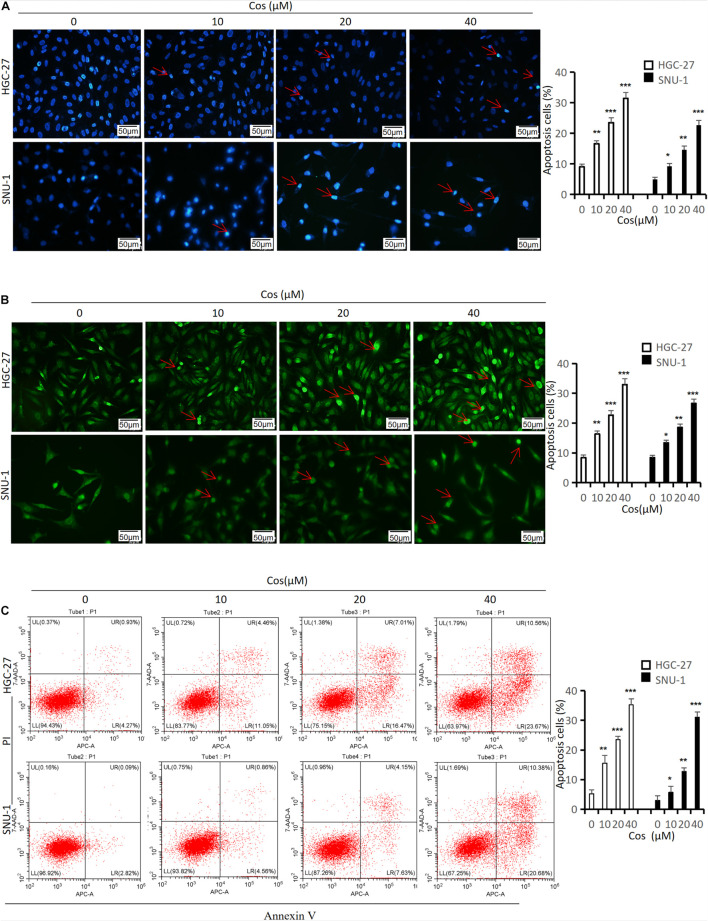
Cos induced apoptosis in GC cells. HGC-27 and SNU-1 were treated with indicated concentration for 24 h. **(A)** The apoptosis-positive cells were stained with Hoechst 33258 staining kit (magnification: × 400) (red arrow = apoptosis cell nucleus). **(B)** The apoptosis-positive cells were stained with Tunel staining kit (magnification: × 400) (red arrow = apoptosis-positive cell). **(C)** HGC-27 and SNU-1 apoptosis was determined by flow cytometry analysis. *p < 0.05, **p < 0.01, ***p < 0.001.

Hoechst 33258, Tunel staining, and flow cytometry were used to evaluate the effect of Cos on apoptosis in GC cells. Hoechst 33258 and Tunel staining showed that along with Cos concentration increase, the rate of apoptosis cell increased (*p* < 0.001) (Figures 3A,B). The flow cytometry results revealed that Cos could dose-dependently lead to the apoptosis of GC cell lines HGC-27 and SNU-1 in the Cos treatment compared with the control group (*p* < 0.001) (Figure 3C).

### Costunolide Induced Intrinsic Apoptosis but Not Extrinsic Apoptosis in GC Cells

**FIGURE 4:**
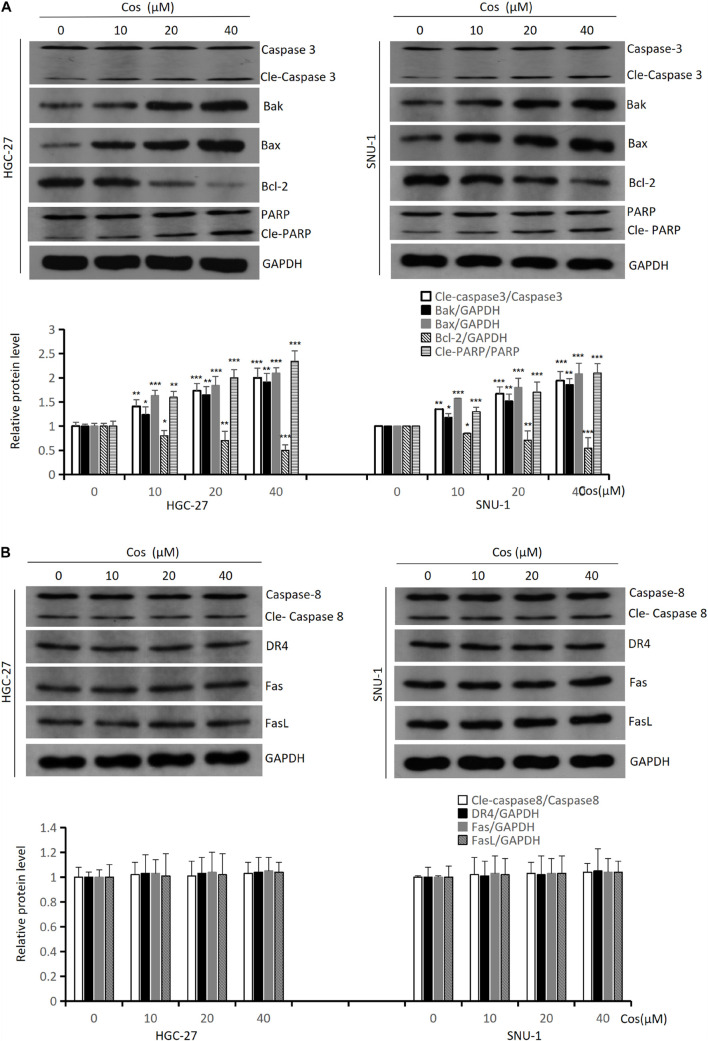
Cos induced intrinsic apoptosis but not extrinsic in GC cells. **(A)** The expressions of the intrinsic apoptosis-related proteins (Caspase 3, Bak, Bax, Bcl-2, and PARP) of Cos induced in HGC-27 and SNU-1 cells were analyzed by Western blot. **(B)** The expressions of the extrinsic apoptosis-related proteins (Caspase 8, DR4, Fas, and FasL) of Cos induced in HGC-27 and SNU-1 were analyzed by Western blot. Compared to control group, *p < 0.05, **p < 0.01, ***p < 0.001.

To further explore the mechanism of Cos-inducing apoptosis, we analyzed intrinsic and extrinsic apoptotic. Western blot revealed that the levels of intrinsic apoptotic proteins [Cleaved-Caspase 3 (Cle-Caspase 3), Bax, Bak, Cleaved-PARP (Cle-PARP)] were upregulated with dose-dependency, but Bcl-2 was downregulated in HGC-27 and SNU-1 cells in the Cos treatment compared with the control group (p < 0.001) (Figure 4A). However, the activities of extrinsic apoptosis proteins [Cleaved-Caspase 8 (Cle-Caspase 8), DR4, Fas, FasL] did not change significantly between the Cos treatment group and the control group (*p* > 0.05) (Figure 4B).

### Costunolide Induces Autophagy in GC Cells

**FIGURE 5:**
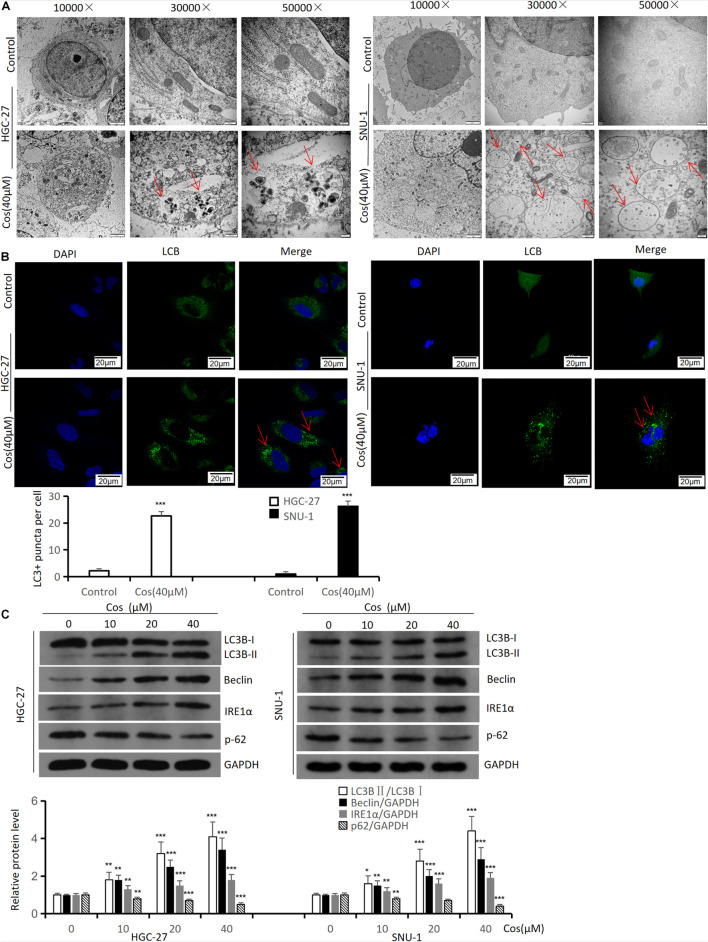
Cos induces autophagy in HGC-27 and SNU-1. HGC-27 and SNU-1 were treated with indicated concentration for 24 h. **(A)** The autophagic microstructures were observed by transmission electron microscopy (red arrow: autophagic vacuole = autophagosome). **(B)** Endogenous LC3 puncta formation was observed by a confocal microscope. **(C)** The protein expression levels of autophagy-related proteins (LC3B, Beclin1, IRE1α, and p62) were analyzed by Western blot. Compared to control group, *p < 0.05, **p < 0.01, ***p < 0.001.

To demonstrate the effect of Cos on autophagy in GC cells, autophagic activity and autophagy-related proteins were analyzed in HGC-27 and SNU-1. Transmission electron microscopy results showed that the formation of autophagic vacuoles in HGC-27 and SNU-1 significantly increased after Cos treatment (Figure 5A). The confocal microscopy results showed that treatment with Cos could lead to the aggregation of autophagosomes both in HGC-27 and SNU-1 (*p* < 0.001) (Figure 5B). Autophagy markers (LC3B, beclin-1, IRE1α) were increased and p62 was decreased after Cos treatment with dose-dependent manner (*p* < 0.001) (Figure 5C).

### Costunolide-Induced Cell Cycle Arrest in GC Cells Was Not via Increasing Reactive Oxygen Species Levels

**FIGURE 6:**
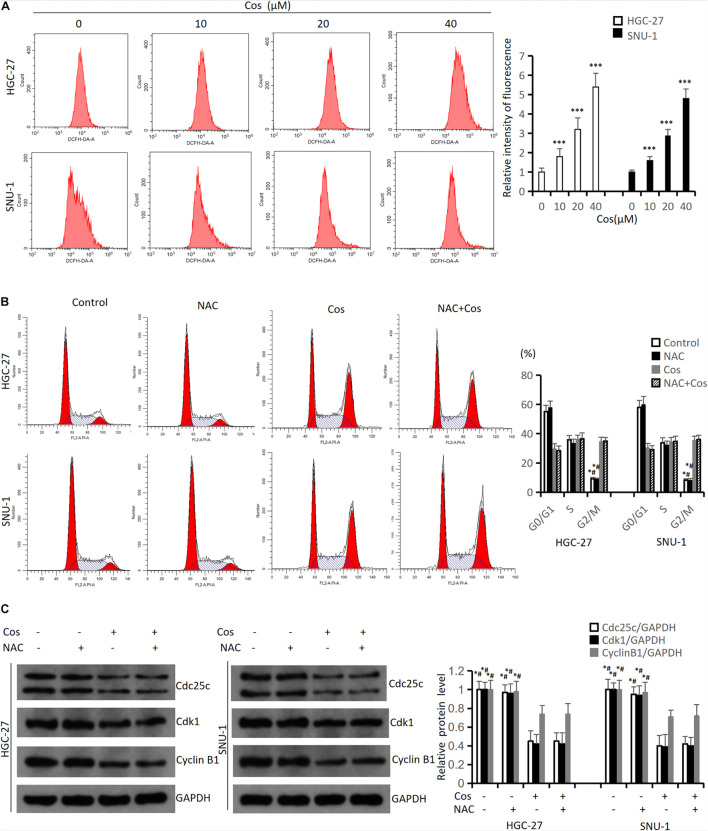
Cos-induced cell cycle arrest in GC cells was not via increasing ROS level. **(A)** HGC-27 and SNU-1 were treated with indicated concentration for 24 h. ROS levels were analyzed using flow cytometry analysis. Compared to control group, ^∗^*p* < 0.05, ^∗∗∗^*p* < 0.001. **(B)** HGC-27 and SNU-1 were pretreated with NAC for 1 h, then treated with indicated concentration for 24 h; cell cycle was analyzed using flow cytometry analysis. Compared to Cos (40 μM), ^∗^*p* < 0.05; compared to NAC + Cos (40 μM), ^#^*p* < 0.05. **(C)** Cell cycle-related proteins was analyzed using Western blot. Compared to Cos (40 μM), ^∗^*p* < 0.05; compared to NAC + Cos (40 μM), ^#^*p* < 0.05.

To investigate the mechanism of Cos-induced cell cycle arrest, apoptosis, and autophagy of GC cell, the levels of ROS were detected. The flow cytometry results showed that Cos could boost ROS generation both in HGC-27 and SNU-1 cells in a dose-dependent manner compared with the control group (*p* < 0.001) (Figure 6A). HGC-27 and SNU-1 cells were first treated with 4 mmol/L NAC (an ROS scavenger) before the cells being incubated with 40 μM Cos. The flow cytometry results showed NAC could not reverse G2/M arrest (*p* > 0.05) (Figure 6B), and the results of cell cycle protein markers in HGC-27 and SNU-1 also showed the same trend (*p* > 0.05) (Figure 6C).

### Costunolide Induced Apoptosis and Autophagy of GC Cell via Increasing Reactive Oxygen Species Level

**FIGURE 7:**
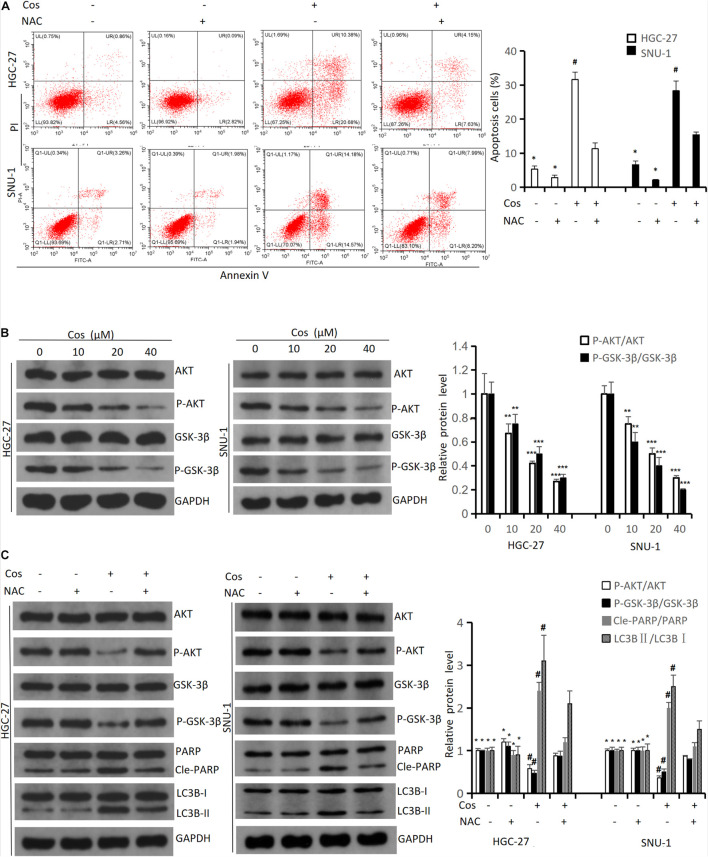
Cos induced apoptosis and autophagy of GC cell via increasing ROS level. **(A)** HGC-27 and SNU-1 were pretreated with NAC for 1 h, then treated with indicated concentration for 24 h; cell apoptosis levels were analyzed using flow cytometry analysis. Compared to Cos (40 μM), *p < 0.05; compared to NAC + Cos (40 μM), ^#^*p* < 0.05. **(B)** HGC-27 and SNU-1 treated with indicated concentration for 24 h, the expressions of signaling pathway-related proteins (AKT, P-AKT, GSK-3β, P-GSK-3β) were analyzed using Western blot. Compared to control group, *p < 0.05, **p < 0.01, ***p < 0.001. **(C)** HGC-27 and SNU-1 were pretreated with NAC for 1 h, then treated with indicated concentration for 24 h, the expressions of signaling pathway-related proteins (AKT, P-AKT, GSK-3β, P-GSK-3β), apoptosis-related protein (PARP), and autophagy-related protein (LC3B) were analyzed using Western blot. Compared to Cos (40 μM), *p < 0.05; compared to NAC + Cos (40 μM), ^#^*p* < 0.05.

The flow cytometry results indicated NAC could significantly reduce Cos-induced apoptosis (*p* < 0.001) (Figure 7A). The Western blot results showed that the ratio of P-AKT/AKT and P-GSK3β/GSK3β markedly downregulated in the Cos treatment groups with dose-dependent manner compared with the control group (*p* < 0.001) (Figure 7B). GC cells were pretreated with 4 mmol/L NAC for 1 h before the cells were treated with 40 μmol/L Cos for 24 h. The ratio of P-AKT/AKT and P-GSK3β/GSK3β in the Cos and NAC co-treated group markedly upregulated higher than that of Cos alone (*p* < 0.05) but downregulated lower than that of NAC alone. In addition, apoptosis-associated protein PARP and autophagy-associated protein LC3BII in the Cos and NAC co-treated group downregulated higher than that of Cos alone (*p* < 0.05) (Figure 7C).

### Costunolide-Induced Cell Cycle Arrest in GC Cells Was Not via Inhibiting AKT/GSK3β Signaling Pathway but Induced Apoptosis and Autophagy via Inhibiting AKT/GSK3β Signaling Pathway

**FIGURE 8:**
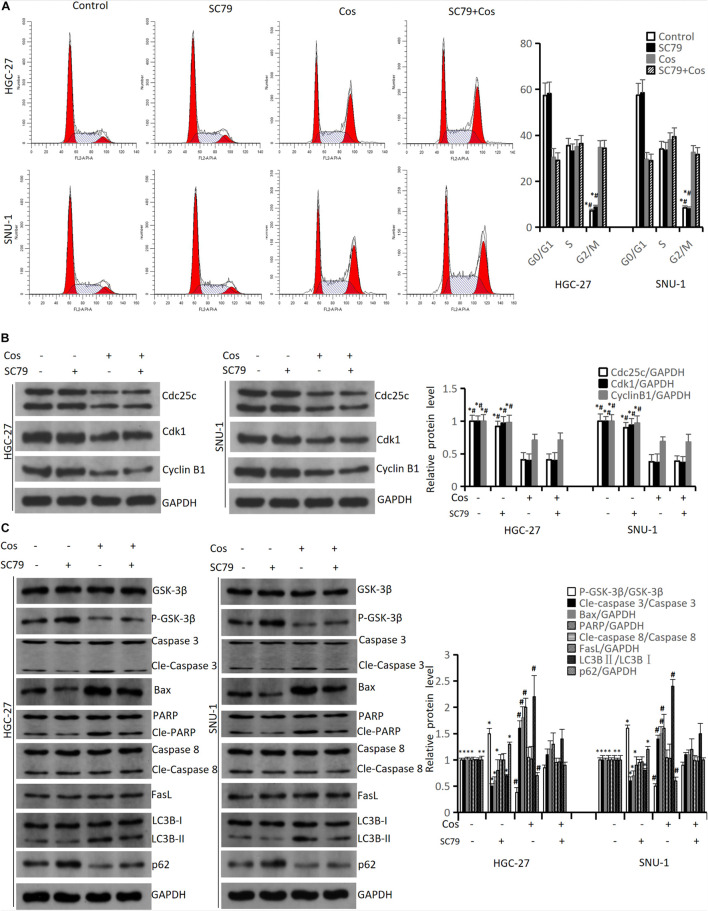
Cos-induced cell cycle arrest in GC cells was not via inhibiting AKT/GSK3β signaling pathway but induced apoptosis and autophagy via inhibiting AKT/GSK3β signaling pathway. HGC-27 and SNU-1 were pretreated with SC79 for 1 h, then treated with indicated concentration for 24 h. **(A)** Cell cycle was determined by flow cytometry analysis. **(B)** The levels of cell cycle-associated proteins were determined by Western blot. **(C)** The expressions of signaling pathway-related proteins (GSK-3β, P-GSK-3β), intrinsic apoptosis-related proteins (Caspase 3, Bax, PARP), extrinsic apoptosis-related proteins (Caspase 8, FasL) and autophagy-related proteins (LC3B, p62) were analyzed using Western blot. Compared to Cos (40 μM), ^∗^*p* < 0.05; compared to SC79 + Cos (40 μM), ^#^*p* < 0.05.

The flow cytometry results revealed SC79 (an AKT activator) could not reverse G2/M arrest in HGC-27 and SNU-1 cells treated with Cos (*p* > 0.05) (Figure 8A), and the results of cell cycle protein markers in HGC-27 and SNU-1 also showed the same trend (*p* > 0.05) (Figure 8B). However, SC79 could reverse apoptosis and autophagy in HGC-27 and SNU-1 cells (*p* < 0.05) (Figure 8C).

### Costunolide Induced Apoptosis via Activating Pro-death Autophagy

**FIGURE 9:**
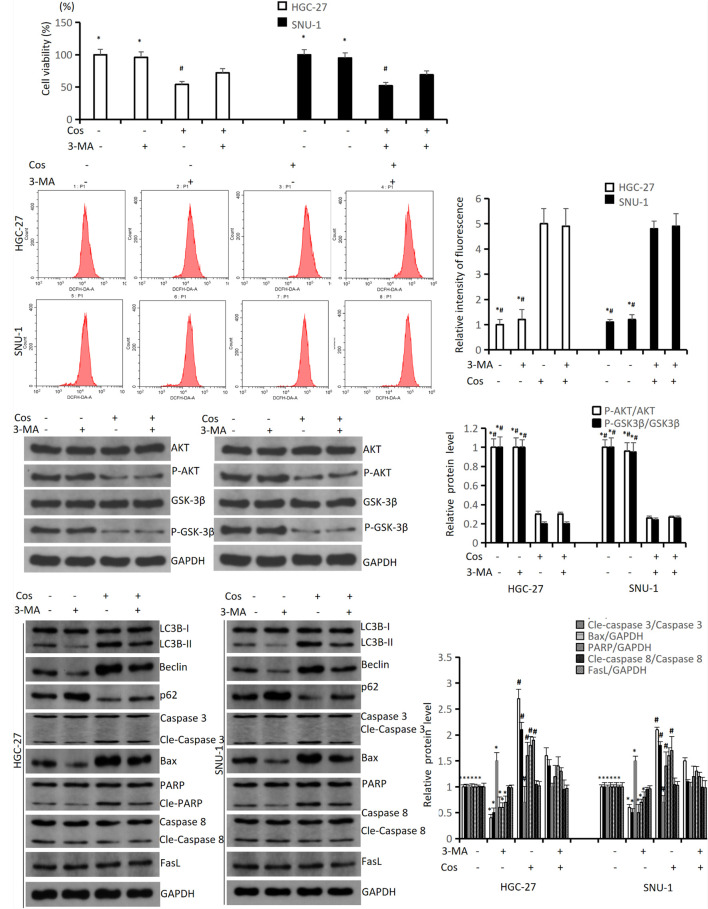
Cos induced apoptosis via activating pro-death autophagy. HGC-27 and SNU-1 were pretreated with 3-MA for 1 h, then treated with indicated concentration of Cos for 24 h. **(A)** The cell viability was analyzed using CCK-8 assay. **(B)** ROS levels were analyzed using flow cytometry analysis. **(C)** The expressions of pathway-related proteins were analyzed using Western blot analysis. **(D)** The expressions of autophagy-related proteins (LC3B, Beclin1, p62), intrinsic apoptosis-related proteins (Caspase 3, Bax, PARP), and extrinsic apoptosis-related proteins (Caspase 8, FasL) were analyzed using Western blot. Compared to Cos (40 μM), ^∗^*p* < 0.05; compared to 3-MA + Cos (40 μM), ^#^*p* < 0.05.

To study the relationships between autophagy and ROS-AKT/GSK3β pathway, and between Cos-induced apoptosis and autophagy, we pretreated HGC-27 and SNU-1 with 4 mmol/L 3-MA (an autophagy inhibitor) for 1 h before the cells were incubated with 40 μM Cos. The results revealed 3-MA could reverse the downregulation of cell viability after Cos treatment in HGC-27 and SNU-1 cells (Figure 9A). The flow cytometry results showed that 3-MA did not reverse the upregulation of ROS after Cos treatment in HGC-27 and SNU-1 cells (Figure 9B), and the Western blot results showed 3-MA also did not reverse the upregulation of P-AKT and P-GSK3β (Figure 9C), which meant autophagy was downstream to ROS-AKT/GSK3β pathway. Western blot results showed that 3-MA could reverse the upregulation of autophagy-related and intrinsic apoptosis-related proteins after Cos treatment in HGC-27 and SNU-1, while extrinsic apoptosis-related proteins were not significantly altered among these groups. This indicated that Cos induced intrinsic apoptosis via activating pro-death autophagy (*p* < 0.05, Figure 9D).

### Costunolide Inhibited Tumor Growth *in vivo*

**FIGURE 10:**
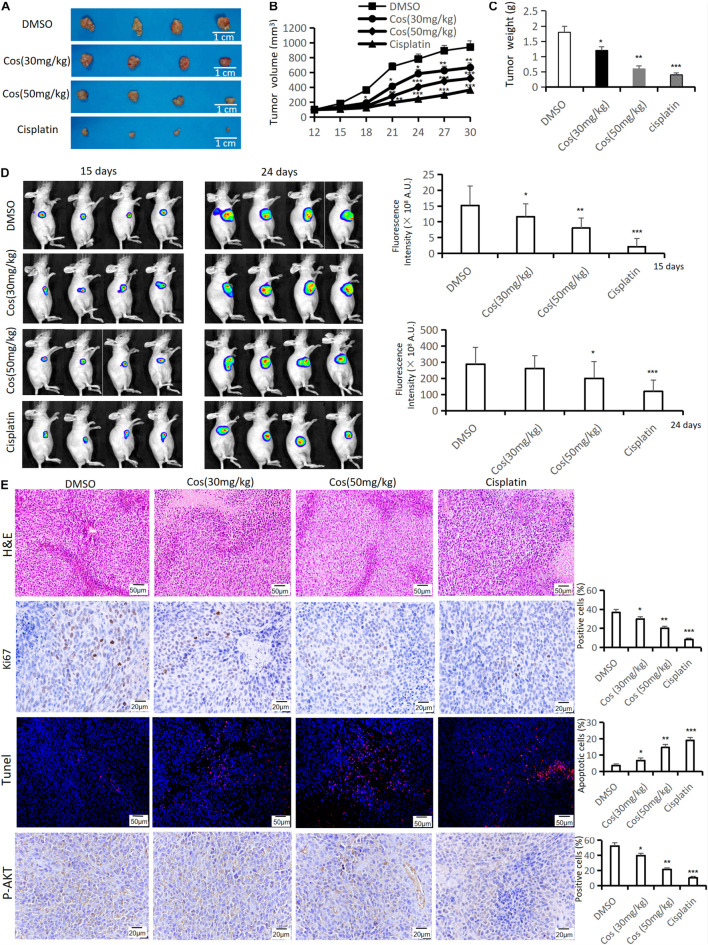
Cos inhibited tumor growth *in vivo*. **(A)** Tumor was taken after 30-day treatment in the DMSO, Cos (30 mg/kg), Cos (50 mg/kg), and cisplatin groups. **(B)** Tumor volume of mice was measured every 3 days. **(C)** Tumor weight of mice was measured after 30-day treatment in the DMSO, Cos (30 mg/kg), Cos (50 mg/kg), and cisplatin groups. **(D)** IVIS images of mice tumor after 15 and 24 days in the DMSO, Cos (30 mg/kg), Cos (50 mg/kg), and cisplatin groups. **(E)** Histochemical analysis of H&E staining, Ki-67, tunel, and P-AKT levels in tumor tissue sections in the DMSO, Cos (30 mg/kg), Cos (50 mg/kg), and cisplatin groups (magnification was ×200, ×400, ×200, and ×400, respectively). *p < 0.05, **p < 0.01, ***p < 0.001.

To estimate the anti-tumor growth effect of Cos *in vivo*, HGC-27 tumor-bearing xenograft nude mouse models were established and treated. The results showed that tumor volume and weight in 30 mg/kg and 50 mg/kg Cos were significantly reduced compared with the DMSO group, especially in the 50 mg/kg group (*p* < 0.01), but both of them increased compared to the Cisplatin group (Figures 10A–C). IVIS images showed the same change after Cos treatment for 15 and 24 days (*p* < 0.01) (Figure 10D). In addition, the HE staining results of tumor tissue revealed the number of tumor cells in tissue sections was decreased by Cos administration in mice and was even less in the 50 mg/kg Cos group. As shown in Ki-67 and P-AKT immunohistochemical staining results, Ki-67 and P-AKT positive ratios were obviously inhibited in the 30 mg/kg and 50 mg/kg Cos group, especially in the 50 mg/kg group, compared with the DMSO group (*p* < 0.01). In contrast, the Tunel staining was increased in Cos-treated mice, especially in the 50 mg/kg Cos group (*p* < 0.01) (Figure 10E).

### Costunolide Induced Apoptosis and Autophagy *in vivo*

**FIGURE 11:**
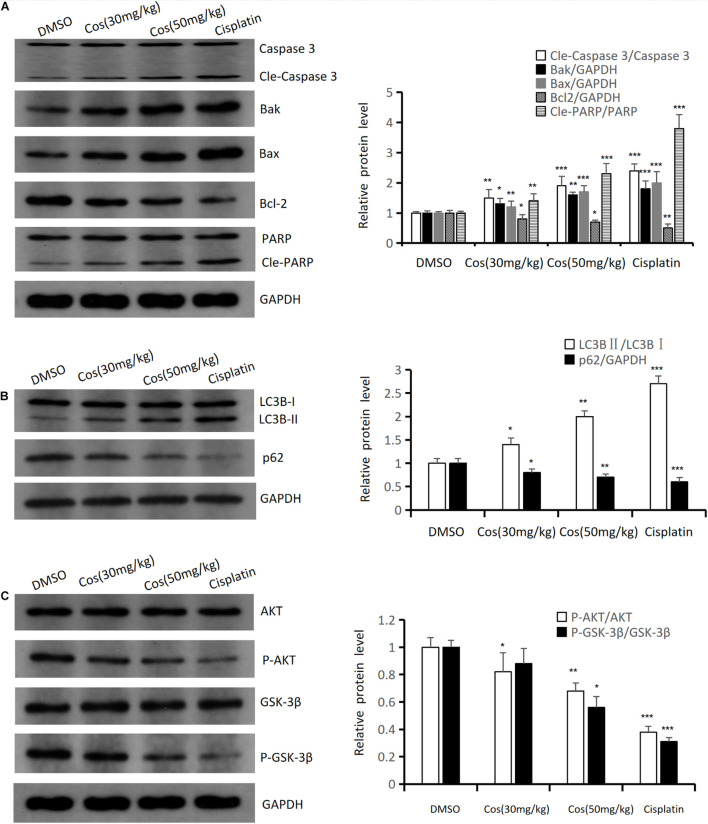
Cos induced apoptosis and autophagy *in vivo*. **(A)** The expressions of apoptosis-related proteins (Caspase 3, Bak, Bax, Bcl-2, and PARP) of Cos induced in mice tumor were analyzed by Western blot. **(B)** The expressions of autophagy-related protein (LC3B, p62) of Cos induced in mice tumor were analyzed by Western blot. **(C)** The expressions of signaling pathway-related protein (AKT, P-AKT, GSK-3β, P-GSK-3β) of Cos induced in mice tumor were analyzed by Western blot. Compared to DMSO group, *p < 0.05, **p < 0.01, ***p < 0.001.

Western blot results confirmed that intrinsic apoptotic associated proteins (Cle-Caspase 3, Bak, Bax, Cle-PARP) (Figure 11A) and autophagy-associated protein LC3BII (Figure 11B) were upregulated in Cos treatment groups, and was higher in the 50 mg/kg Cos-treated group compared with DMSO group, while apoptosis-related protein Bcl-2, autophagy-related protein p62, and the ratio of P-AKT/AKT and P-GSK3β/ GSK3β (Figure 11C) were significantly decreased in the Cos treatment group, especially in the 50 mg/kg treatment group, compared with DMSO group (*p* < 0.001).

### Costunolide Had No Side Effects in Major Organs *in vivo*

**FIGURE 12:**
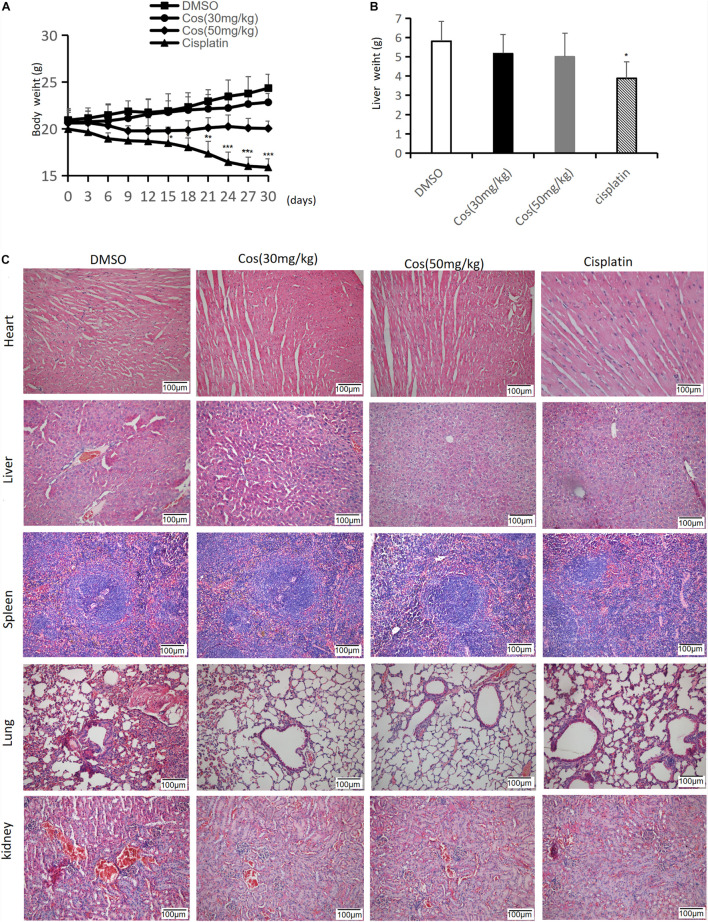
Cos had no side effects in major organs *in vivo*. **(A)** The body weight of mice was measured every 3 days. **(B)** Liver weight of mice was measured after 30 days. **(C)** H&E staining of heart, liver, spleen, lung and kidney tissue sections (magnification: ×200) was measured after Cos treatment for 30 days. Compared to the DMSO group, *p < 0.05, **p < 0.01, ***p < 0.001.

The results showed no significant change in body and liver weight between the Cos treatment group and the DMSO group (*p* > 0.05) (Figures 12A,B), and HE staining of pathological sections elucidated that Cos treatment had no evident damage to the major organs (heart, liver, spleen, lung, and kidney) of mice (Figure 12C), which confirmed the safety of Cos *in vivo*.

## Discussion

With the advancement of medical technology, the therapy of gastric cancer has improved to a certain extent. However, due to the side effects and damage of radiotherapy and chemotherapy, the 5-year survival rate is still very poor (Bray et al., 2018). Therefore, more effective therapeutic methods and drugs are urgently required. In recent years, natural plant-derived ingredients have been widely applied in the medical field due to their low toxicity and various biological activities (Yu et al., 2017). In China, natural products, such as artemisinin (qinghaosu), have been universally applied in the treatment of malaria for long history (Tu, 2011). Consequently, natural products have been regarded as pioneers in drug discovery (Mosca et al., 2020).

Cos is a naturally active sesquiterpene lactone extracted from the medicinal plant and possesses remarkable and diverse biological and immunological properties, such as anti-cancer, anti-microbial, and neuroprotective activities (Kim and Choi, 2019; Peng et al., 2019; Liu et al., 2020), a key medicine for treating various gastrointestinal disorders (Wang W. et al., 2020). As we all know, there are many risk factors for gastric cancer, containing gastric ulcer, atrophic gastritis, and *Helicobacter pylori* infection (Park et al., 1997). Cos can resist these risk factors (Xie et al., 2020), which is particularly important in the prevention and adjuvant treatment of gastric cancer. Some researches revealed that Cos exerted anti-tumor activity by suppressing cell proliferation. One research indicated that Cos prevented the proliferation of liver cancer cells by regulating the signaling pathway of epithelial growth factor (EGF) (Si et al., 2020). Another reported that Cos inhibited the proliferation, invasion, and metastasis of osteosarcoma by inhibiting the STAT3 signaling pathway (Jin et al., 2020). Moreover, Cos suppressed the proliferation in leukemic cell (Saosathan et al., 2021) and ovarian cancer cells (Fang et al., 2019). We discovered Cos inhibited the proliferation of gastric carcinoma cells, and the inhibitory effect of Cos specifically targets gastric cancer cells because Cos has no obvious inhibitory effect on normal gastric mucosal GES-1 cells, and Cos induced cell cycle arrest in GC cells but has no obvious effect on GES-1 cell. The effectiveness and safety of Cos was also verified in an animal model, with evidence confirming that in body and liver weight, there was no significant difference between the Cos treatment group and Control group. However, we just used one normal gastric mucosal cell line GES-1 in our study. In future experiments, we will obtain a couple of other normal gastric mucosal cells lines as control group, which will be more convincing. Studies have found that Cos inhibits the proliferation of human ovarian cancer cells via activating apoptosis and autophagy (Fang et al., 2019). Moreover, in renal cell carcinoma, Cos also caused apoptosis and autophagy via triggering ROS/MAPK signaling pathways (Fu et al., 2020). A previous study revealed Cos-induced apoptosis in human gastric cancer cells, but the autophagy activity and the relationship between apoptosis and autophagy of Cos induced in gastric cancer are seldom studied. This study found that Cos could significantly inhibit HGC-27 and SNU-1 growth, induce G2/M phase arrest, and trigger apoptosis and autophagy in a dose-dependent manner. Further experiments confirmed that Cos improved cellular ROS levels and blocked the AKT/GSK3β signaling pathway. NAC pretreatment reversed the effects of Cos-induced apoptosis and autophagy via AKT/GSK3β signaling activation. Moreover, Cos induced pro-death autophagy to activate apoptosis.

Deregulation of the cell cycle represents an important trait of tumors (Yu et al., 2020). Many anti-cancer drugs inhibit tumor cell proliferation via stalling the cell cycle (Wu et al., 2020). Cos was found to induce G1/S phase arrest in human esophageal carcinoma Eca-109 cells (Hua et al., 2016b) and induce G2/M phase arrest in human liver cancer HepG2 cells and breast cancer MDA-MB-231 cells (Mao et al., 2019). Our study revealed Cos could significantly induce GC cell cycle arrest in the G2/M phase via mediated Cyclin B1, Cdc25c, and Cdk1 protein expression.

Another trait of tumors is their ability to evade apoptosis. Therefore, inducing apoptosis represents an indispensable mechanism for anti-cancer drugs (Zhang et al., 2016; Kang et al., 2019; Liu et al., 2019). Cos was previously confirmed to induce apoptosis in human gastric carcinoma, prostate cancer, liver cancer, bladder cancer, and esophageal carcinoma. In accordance with these findings, our study indicated that Cos could induce the apoptosis of gastric cancer cell lines HGC-27 and SNU-1. Drugs induce cancer cell apoptosis through the mitochondrial or the extrinsic apoptosis pathway depending on the type of cancer cell and other factors. Recent studies indicated that Cos induces cell apoptosis of bladder cancer and lung cancer via mitochondrial pathways and induces leukemia cancer and breast cancer via extrinsic pathways (Hua et al., 2016a; Zhang et al., 2016; Hu et al., 2018). Our results showed that Cos upregulated mitochondrial apoptosis protein expression of Caspase 3 and PARP, and the ratio of Bax/Bcl-2 and Bak/Bcl-2. However, extrinsic apoptosis proteins [Cle-Caspase 8, DR4, Fas, Fas ligand (FasL)] were not significantly altered, suggesting that Cos induced apoptosis via intrinsic (mitochondrial) pathway in gastric cancer cells.

Autophagy is a lysosomal degradation pathway with the characterization of an increase in the number of acidic vesicle organelles associated with autophagosomes, dysregulating in cancer cells as another important way of PCD (Kanno et al., 2008; Choi et al., 2012). Autophagy has the dual effects of promoting cell death and inhibiting cell death, depending on tumor cell types (Yun and Lee, 2018). Recent studies exhibited that Cos could activate autophagy in renal cell carcinoma and ovarian cancer through the ROS/MAPK pathway (Fu et al., 2020), while inhibiting autophagy in hepatocellular carcinoma cells (Okubo et al., 2021). Results of this study confirmed that Cos significantly activated autophagy, featured by the increased expression of LC3BII and Beclin 1, while p62 decreased in a dose-dependent manner. That was contradictory to the report that apigenin could induce autophagy and promote the increase in p62 expression (Wei et al., 2020), but consistent with the report that Tanshinone I activated autophagy via decreasing the expression of p62 (Zhou et al., 2020). The reason for the p62 decrease in our study may be that p62 protein is located on the autophagosome by LC3 binding, and it is degraded by autophagy (Dong et al., 2020).

Reactive oxygen species are by-products of aerobic metabolism. Higher ROS levels are observed in various cancer cells than normal cells (Gorrini et al., 2013), and ROS is a vital factor for drug-activated apoptosis and autophagy (Zhang et al., 2016). Cos induced apoptosis through ROS-mediated endoplasmic reticulum stress in human U2OS cells (Wang et al., 2016). Cos also increased ROS levels in human esophageal carcinoma Eca-109 cells, lung adenocarcinoma A549 cells, and renal cell carcinoma, leading to apoptosis and autophagy (Nadda et al., 2020). Cos could dose-dependently promote ROS generation in gastric cancer cells, and NAC pretreatment could reverse Cos-induced apoptosis and PARP spliceosome generation. As an important effector downstream of ROS, AKT/GSK3β mediates the apoptosis and autophagy of a variety of cells (Deng et al., 2019; Wang et al., 2019; Zhao et al., 2019). One study reported that it suppresses gastric cancer by repressing AKT/GSK3β signaling to inhibit autophagy (Dai et al., 2021). Another reported placenta-specific 8 inhibited oral squamous cell carcinogenesis via blocking AKT/GSK3β signaling pathways (Wu et al., 2020). This study confirmed the inhibitory effects of Cos on the AKT/GSK3β pathway, which was reversed by SC79 (AKT activator) pretreatment. These results indicate that Cos promoted autophagy and apoptosis via inhibiting the ROS-mediated AKT/GSK3β pathway in HGC-27 and SNU-1, which is consistent with animal experiment results.

At last, we also proved that Cos activated prodeath autophagy to induce intrinsic apoptosis via modulation of the AKT/GSK-3β signaling pathway in gastric cancer (Figure 12). The mechanism has been further confirmed that the Cos plus 3-MA (an inhibitor of autophagy) treatment significantly inhibited the expression level of apoptosis-related proteins compared with Cos alone. It was reported that the overexpression of p62 could promote cell apoptosis, which is related to the ubiquitin-associated (UBA) domain at the C terminal (Zhang et al., 2013). This finding indicates that p62 protein can be used not only as a marker for autophagy activation but also as an important regulator of apoptosis.

**FIGURE 13:**
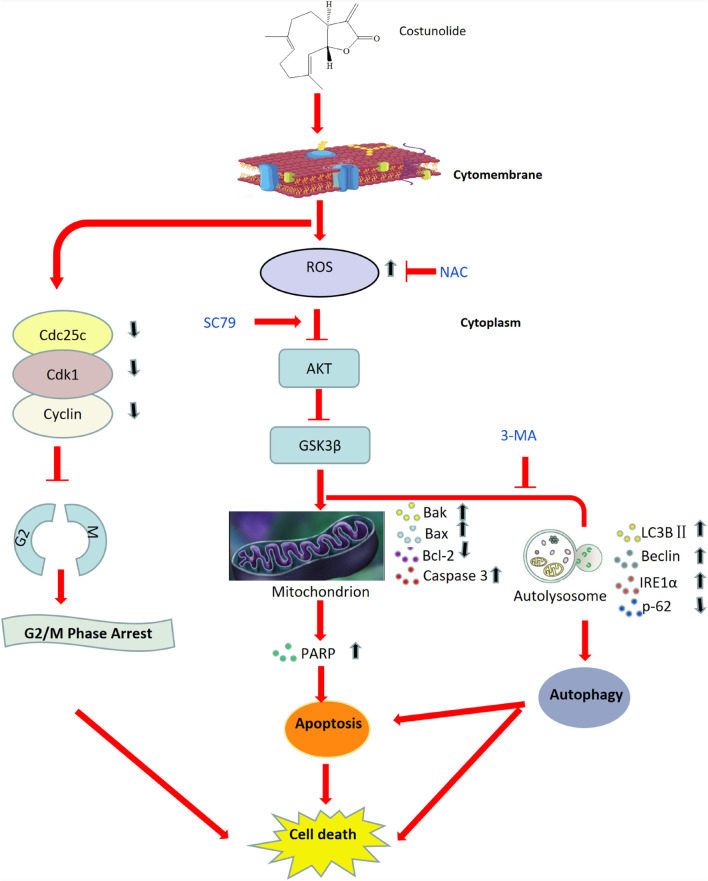
Working model illustrating the anti-tumor mechanism of Cos in gastric cancer.

In summary, Cos significantly inhibited cell proliferation, hindered G2/M phase progression, and promoted apoptosis and autophagy in HGC-27 and SNU-1. Mechanistic studies reveal that Cos promoted ROS generation and inhibited the AKT/GSK3β pathway, thus triggering cell-intrinsic apoptosis through activating prodeath autophagy (Figure 13). This study showed that Cos might be a potential drug for the treatment of gastric cancer. However, there were some limitations in our study. Firstly, we just chose the female Balb/c nude mice for an animal model; it may be a limitation. In the future, we will use a mix of sexes for animal studies. In addition, in this present study, we only used small-molecule inhibitors as methods of perturbation, such as NAC, SC79, and 3-MA. In the following experiment, we will include orthogonal approaches such as siRNA-mediated knockdown or gene overexpression to confirm the results. Lastly, in order to further investigation in Cos development, we will strictly design the clinical trial program and perform rigorous clinical trials with actual tumor level data to clarify.

## Data Availability Statement

The datasets presented in this study can be found in online repositories. The names of the repository/repositories and accession number(s) can be found in the article/Supplementary Material.

## Ethics Statement

The animal study was reviewed and approved by Biomedical Ethics Committee of Shaanxi Provincial People’s Hospital.

## Author Contributions

CX, XH, JW, and ZJ conceived and designed the experiments. CX, XH, MW, YH, XLi, YH, XZ, JS, XD, and ZJ performed the experiments. CX, XH, and JW analyzed the data. CX, XH, XLe, and YX made data interpretation and critical manuscript revisions. CX, XH, and ZJ wrote the manuscript. All the authors have read and approved the final manuscript.

## Conflict of Interest

The authors declare that the research was conducted in the absence of any commercial or financial relationships that could be construed as a potential conflict of interest.

## Publisher’s Note

All claims expressed in this article are solely those of the authors and do not necessarily represent those of their affiliated organizations, or those of the publisher, the editors and the reviewers. Any product that may be evaluated in this article, or claim that may be made by its manufacturer, is not guaranteed or endorsed by the publisher.

## Supplementary Material

Supplementary Figure 1 Cos did not induced apoptosis and autophagy in GES-1 cells, and has no effect on GSK3β pathway.

Supplementary Figure 2 3-MA reversed the upregulation of apoptosis after Cos treatment in GC cells. Compared to Cos (40 μM), **P* < 0.05; Compared to 3-MA + Cos (40 μM), ^#^*P* < 0.05.

Supplementary Figure 3 Cos inhibited tumor growth *in vivo*. Tumor is taken after 30 days treatment in DMSO, Cos (30 mg/kg), Cos (50 mg/kg), cisplatin group.
