# Supplementary figures and images for: Costunolide-Induced Apoptosis via Promoting the Reactive Oxygen Species and Inhibiting AKT/GSK3β Pathway and Activating Autophagy in Gastric Cancer
Source: Front Cell Dev Biol. 2021 Nov 12;9:722734. doi: 10.3389/fcell.2021.722734 (PMC8633576; doi:10.3389/fcell.2021.722734)

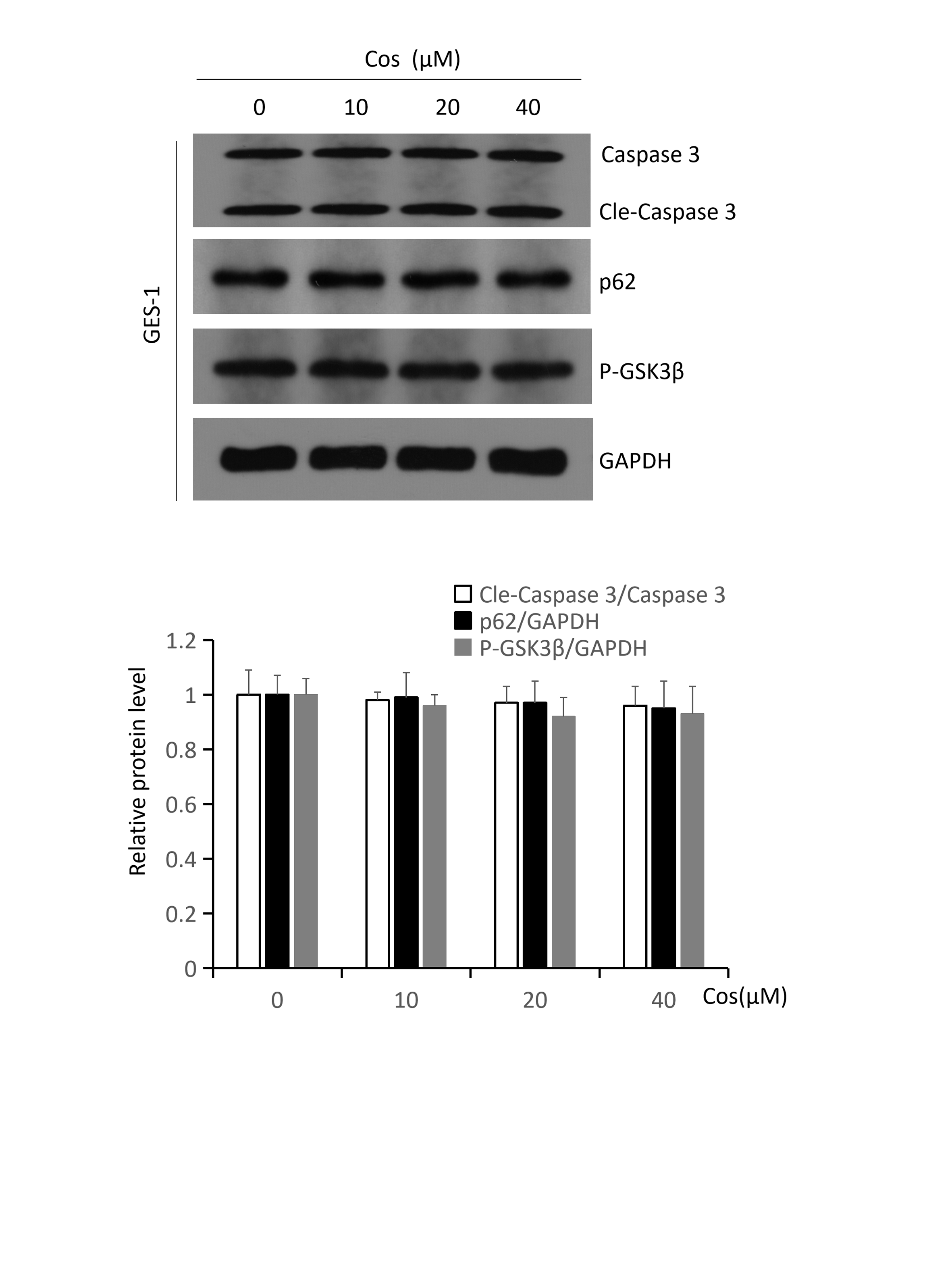

Supplement: Supplementary Figure 1 — Cos did not induced apoptosis and autophagy in GES-1 cells, and has no effect on GSK3β pathway. [file Image_1.TIF]

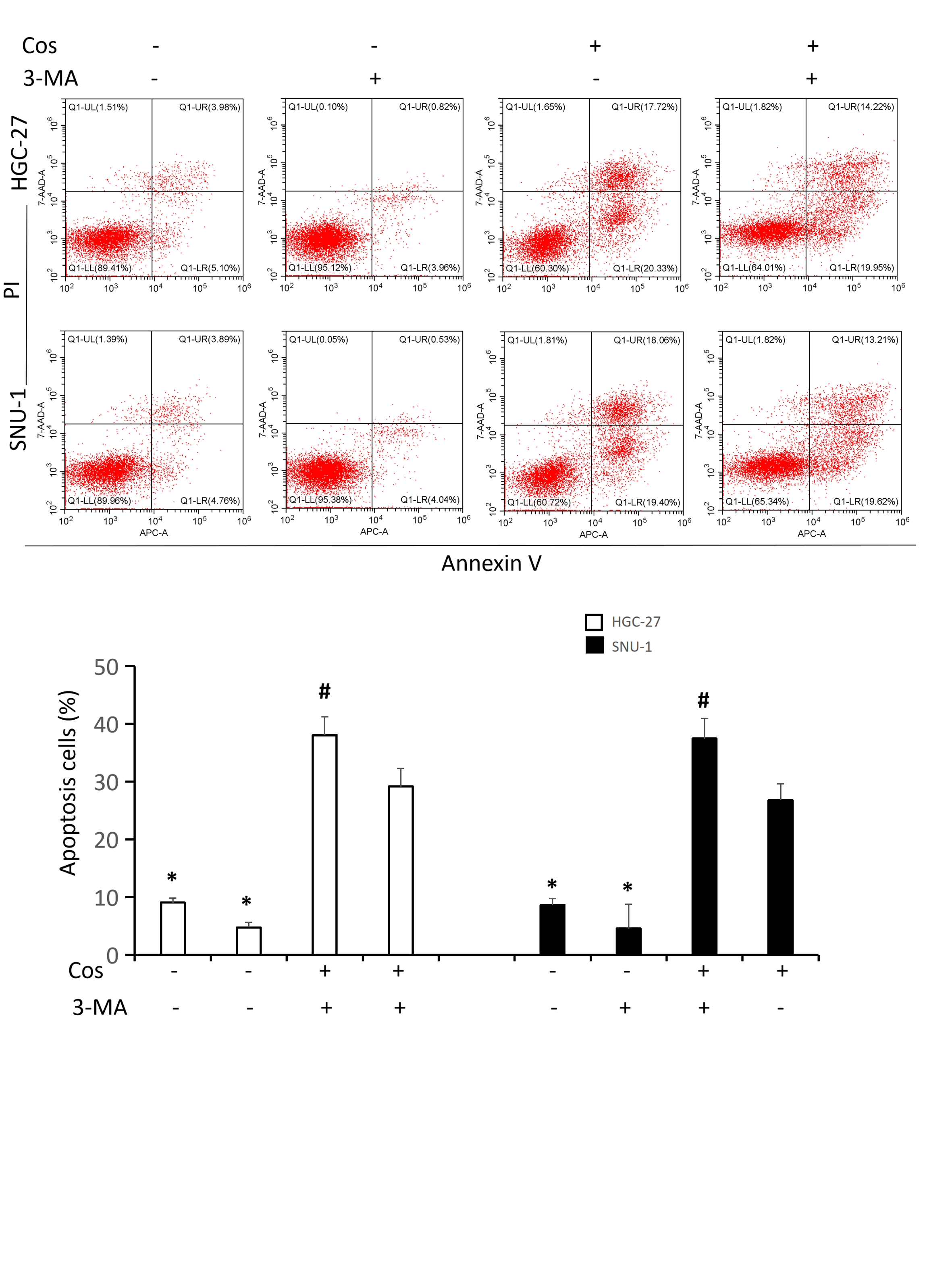

Supplement: Supplementary Figure 2 — 3-MA reversed the upregulation of apoptosis after Cos treatment in GC cells. Compared to Cos (40 μM), *P < 0.05; Compared to 3-MA + Cos (40 μM), #P < 0.05. [file Image_2.TIF]

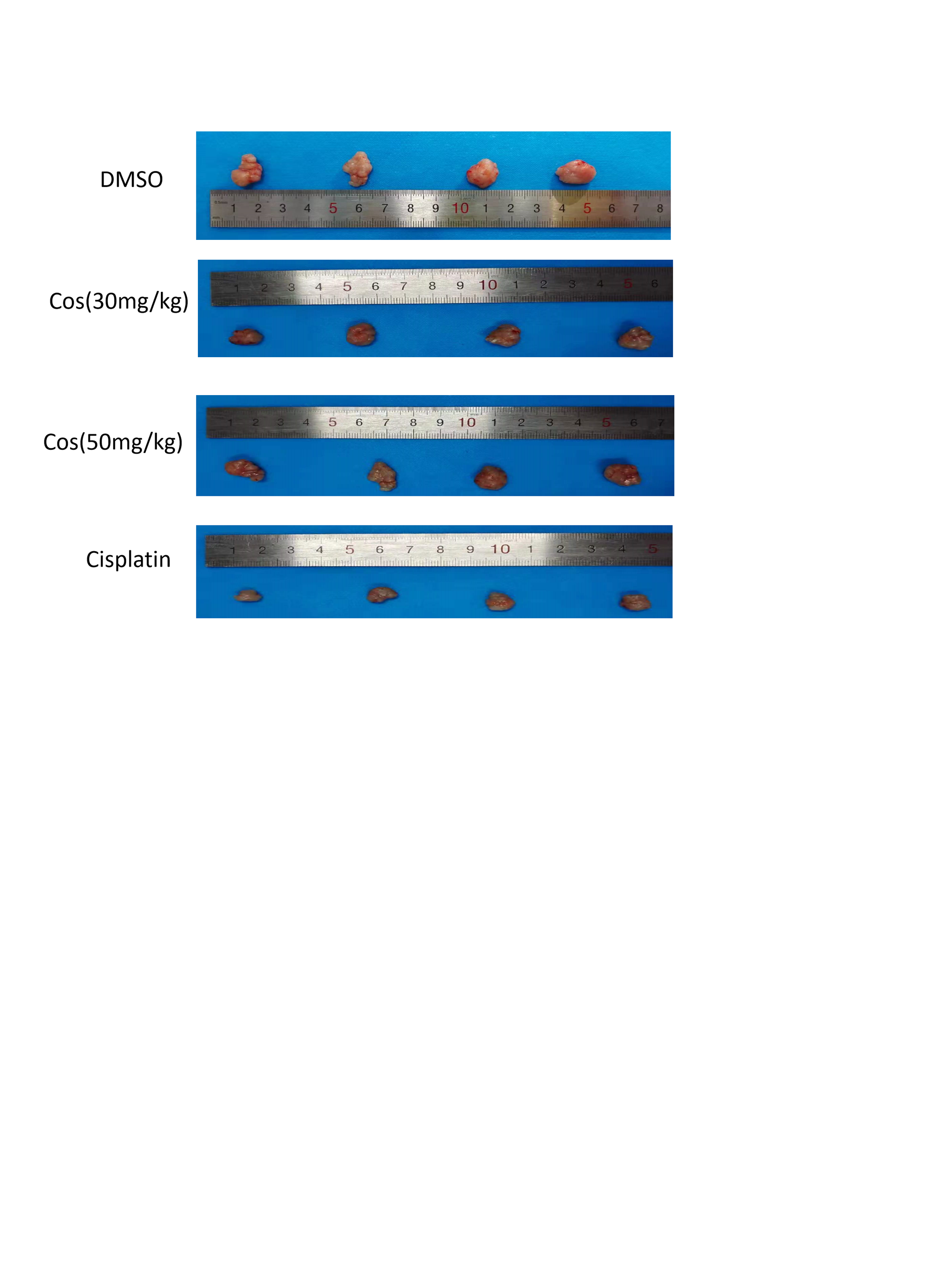

Supplement: Supplementary Figure 3 — Cos inhibited tumor growth in vivo. Tumor is taken after 30 days treatment in DMSO, Cos (30 mg/kg), Cos (50 mg/kg), cisplatin group. [file Image_3.TIF]
